# Antibacterial Activity of Ethanolic Extracts of *Origanum majorana*, *Salvia officinalis*, and *Ribes nigrum* Against Digestive Pathogens: Polyphenolic Composition and In Vitro Assessment

**DOI:** 10.3390/molecules30163341

**Published:** 2025-08-11

**Authors:** Oana-Roxana Haralambie, Cristiana-Ștefania Novac, Dragoș Hodor, Florica Ranga, Sanda Andrei

**Affiliations:** 1 Department of Preclinical Sciences, Faculty of Veterinary Medicine, University of Agricultural Sciences and Veterinary Medicine, 400372 Cluj-Napoca, Romania; 2 Department of Microbiology, Immunology and Epidemiology, Faculty of Veterinary Medicine, University of Agricultural Sciences and Veterinary Medicine, 400372 Cluj-Napoca, Romania; cristiana.novac@usamvcluj.ro; 3 Department of Anatomic Pathology, Faculty of Veterinary Medicine, University of Agricultural Sciences and Veterinary Medicine, 400372 Cluj-Napoca, Romania; dragos.hodor@usamvcluj.ro; 4 Faculty of Food Science and Technology, University of Agricultural Sciences and Veterinary Medicine, 400372 Cluj-Napoca, Romania; floricuta.ranga@usamvcluj.ro

**Keywords:** *Origanum majorana*, *Salvia officinalis*, *Ribes nigrum*, plant extracts, digestive bacteria, antimicrobial activity, polyphenols

## Abstract

Digestive pathologies are commonly encountered in both human and veterinary medicine, frequently requiring antibiotic intervention. However, their extensive use has contributed to the global increase in antimicrobial resistance, posing a major public health challenge. With the emergence of multidrug-resistant bacteria, alternative antimicrobial strategies are urgently needed. This study assessed the total polyphenolic content and in vitro antimicrobial activity of ethanolic extracts from *Origanum majorana*, *Salvia officinalis*, and *Ribes nigrum* fruits against six digestive bacterial pathogens: *Escherichia coli*, *Salmonella enteritidis*, *Enterobacter cloacae*, *Yersinia enterocolitica*, *Listeria monocytogenes*, and *Enterococcus faecalis*. Antimicrobial activity was evaluated using agar well diffusion and minimum inhibitory concentration (MIC) assays. The total polyphenolic content of the extracts was 8509.457 μg/g for Salvia officinalis, 8140.996 μg/g for *Origanum majorana*, and 5776.616 μg/g for *Ribes nigrum*. *R. nigrum* showed the strongest antimicrobial effect (MIC 0.002 μg/μL; MBC 0.001 μg/μL) against *Y. enterocolitica*. *S. officinalis* had the highest efficacy against *E. faecalis*, while *O. majorana* was effective against both *Y. enterocolitica* and *E. faecalis*. All extracts showed bactericidal activity with MIC index values between 0.5 and 4. These findings suggest that these polyphenol-rich plant extracts may serve as promising natural antimicrobials or as adjuvants to conventional antibiotics.

## 1. Introduction

The intestinal microbiota plays a crucial role in maintaining host health by shaping immune system development and enhancing nutrient absorption. Among its key protective functions is colonization resistance, which limits the invasion of external pathogens and controls the growth of potentially harmful native microbes known as pathobionts [1]. The intestinal epithelial barrier further reinforces this defense by segregating the gut microbiota from internal sterile tissues, primarily through tight junctions. These junctions help prevent microbial translocation; however, several enteric pathogens, including *Salmonella*, *Escherichia coli* (enteroinvasive and enteropathogenic strains), and *Yersinia*, have evolved strategies to disrupt this barrier and invade host tissues [2]. Under certain conditions, such as inflammatory bowel disease, the microbiota itself may contribute to disease progression [1]. In parallel, the global rise of multidrug-resistant (MDR) bacteria, mainly due to antibiotic overuse, has intensified the search for alternative treatments [3,4]. In this context, natural products with antimicrobial and antioxidant properties have gained increasing attention. Notably, over 60% of currently used anticancer agents are derived from natural sources [5]. Plant-based medicine continues to be regarded as a safer therapeutic approach, offering a wide range of bioactive compounds with fewer side effects and potential efficacy against complex diseases such as cancer [6].

*Origanum majorana* L. (sweet marjoram), a member of the *Lamiaceae* family, is a perennial herb native to the Mediterranean region and cultivated worldwide. Traditionally used for culinary and medicinal purposes, it contains various active compounds known for their antibacterial, antioxidant, anti-inflammatory, and antifungal properties [3,7].

*Salvia officinalis* (sage), also a member of the *Lamiaceae* family, comprises over 20 medicinal species. It is used worldwide to treat inflammation, infections, and digestive problems [4,5,8]. Sage contains compounds with antibacterial, antioxidant, antitumor, and anti-inflammatory properties. Its anticancer potential is especially noted [5].

*Ribes nigrum* L. (blackcurrant) is rich in anthocyanins, flavonols, and phenolic acids. It has potent antioxidant and antimicrobial properties [9,10]. Studies have shown that consuming blackcurrant-derived products can lead to a decrease in *Bacteroides* and *Clostridium* spp. populations, while promoting the growth of beneficial microbes such as *Bifidobacteria* and *Lactobacillus* [11]. It also shows anticancer activity against colon, breast, and leukemia cells [9]. Blackcurrant supplements are used to boost immunity [12]. They also help balance gut bacteria and reduce cancer-related markers [11]. With the growing threat of antimicrobial resistance, especially in veterinary medicine, where conventional antibiotics are heavily used, there is an increasing demand for effective, plant-based alternatives.

*Origanum majorana* L., *Salvia officinalis*, and *Ribes nigrum* L. fruits are recognized for their abundance of bioactive compounds and significant polyphenol content, which contribute to a wide range of biological effects, including antimicrobial properties. In this context, the present study aims to investigate the in vitro antimicrobial activity of ethanolic extracts from *O. majorana*, *S. officinalis*, and *R. nigrum* fruits against a selection of intestinal pathogenic bacteria.

## 2. Results

### 2.1. Determination of Total Phenolic Content: High-Performance Liquid Chromatography–Diode Array Detection–Electro-Spray Ionization Mass Spectrometry (HPLC-DAD-ESI-MS) for Polyphenols Profile

**Figure 1:**
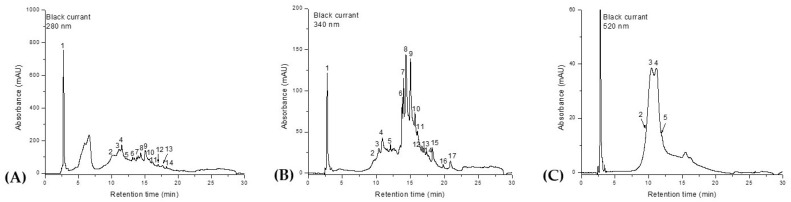
HPLC chromatograms of phenolic compounds of *Ribes nigrum* extract. Identification and quantification of phenolic acids at λ = 280 nm (**A**), flavonoids at λ = 340 nm (**B**), and anthocyanins at λ = 520 nm (**C**). Peaks’ numbers refer to the compounds listed in Table 1.

The HPLC-DAD-ESI^+^ profile of the phenolic composition of *Ribes nigrum* fruit extract is presented in Figure 1 and Table 1. Identification of the phenolic compounds was achieved by comparing the retention times, UV–Vis spectra, and mass spectral data of individual peaks with those reported in the literature.

Out of a total of 17 compounds, only one phenolic acid—2,3-dihydroxybenzoic acid—was identified. The anthocyanin class was represented by delphinidin-glucoside, delphinidin-rutinoside, cyanidin-glucoside, and cyanidin-rutinoside. The most abundant flavonols were kaempferol-rhamnoside, quercetin-diglucoside, myricetin-glucoside, quercetin-rutinoside, quercetin-glucoside, quercetin-rhamnoside, kaempferol-glucoside, isorhamnetin-glucoside, and myricetin-rhamnoside, as well as the aglycones myricetin, quercetin, and kaempferol. From a quantitative perspective, 2,3-dihydroxybenzoic acid was identified as the predominant compound, followed by quercetin-rutinoside (rutin) and myricetin-glucoside. Kaempferol was detected in the lowest concentration.

**Figure 2:**
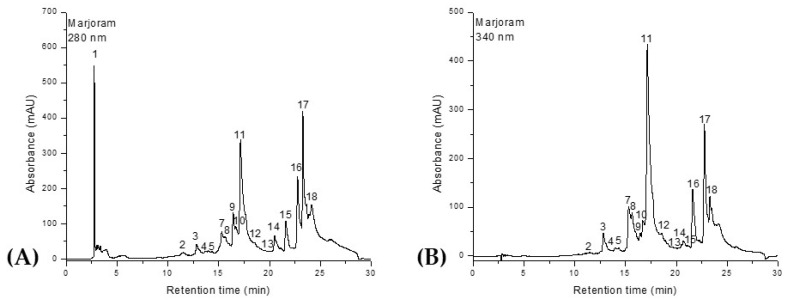
HPLC chromatograms of phenolic compounds in *Origanum majorana* extract. Phenolic acids were identified and quantified at λ = 280 nm (**A**), while flavonoids were detected at λ = 340 nm (**B**). Peak numbers correspond to the compounds listed in Table 2.

**Figure 3:**
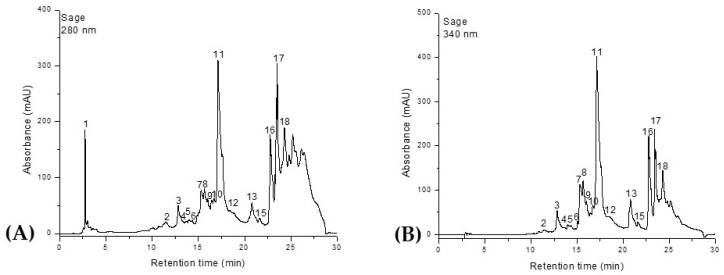
HPLC chromatograms of phenolic compounds in *Salvia officinalis* extract. Phenolic acids were identified and quantified at λ = 280 nm (**A**), while flavonoids were detected at λ = 340 nm (**B**). Peak numbers correspond to the compounds listed in Table 2.

Similarly, the HPLC-DAD-ESI^+^ profiles of the phenolic composition of the *Salvia officinalis* and *Origanum majorana* extracts are shown in Figure 2 and Figure 3 and Table 2.

A total of 18 compounds were identified in the ethanolic extracts of both plants, classified into four distinct phytochemical groups. Phenolic acids were represented by ferulic acid, 5-caffeoylquinic acid (chlorogenic acid), rosmarinic acid, and 2,3-dihydroxybenzoic acid. The flavonol class included quercetin, quercetin-rutinoside (rutin), and quercetin-glucoside (isoquercitrin). Flavones were the most abundant group, comprising luteolin, luteolin-rutinoside, luteolin-glucuronide, luteolin-glucoside, apigenin, apigenin-glucoside, apigenin-acetyl-glucoside, and apigenin-glucuronide. The final group, diterpenes, was represented by carnosic acid, rosmanol, and rosmadial. Although rosmarinic acid is the main compound present in both plants, *Salvia officinalis* contains it in a higher proportion compared to *Origanum majorana*. Ferulic acid is also found in significant amounts in both species, ranking second in terms of quantity. In *O. majorana*, the third most abundant compound is 2,3-dihydroxybenzoic acid, while in S. officinalis this position is occupied by quercetin-glucoside (isoquercitrin). The lowest phenolic content in marjoram was recorded for apigenin-glucoside, whereas in sage it was apigenin-acetyl-glucoside. Additionally, *S. officinalis* contains quercetin-rutinoside (rutin), which was not identified in *O. majorana*. In contrast, *O. majorana* contains quercetin, a compound that was not detected in the *S. officinalis* samples.

Accordingly, analysis of the ethanolic extracts from *Ribes nigrum* fruits, *Salvia officinalis*, and *Origanum majorana* revealed total polyphenolic contents of 5776.616 μg/g, 8509.457 μg/g, and 8140.996 μg/g, respectively.

### 2.2. Antimicrobial Activity by the Agar Well Diffusion Method

Both plants (marjoram and sage), as well as blackcurrant fruits, exhibited in vitro antimicrobial activity against pathogenic intestinal bacteria. In the case of the *Ribes nigrum* extract, the largest inhibition zone was recorded against *Yersinia enterocolitica* (13.24 ± 0.06 mm) among Gram-negative bacteria, showing a comparable effect to the gentamicin control (*p* > 0.05). Moreover, the extract demonstrated promising antimicrobial activity against *Escherichia coli* (13.00 ± 0.03 mm), followed by *Enterobacter cloacae* (12.34 ± 0.01 mm). The smallest inhibition zone was observed for *Salmonella enteritidis* (9.57 ± 0.04 mm). However, all inhibition zones mentioned above were significantly smaller than those produced by the gentamicin control (*p* < 0.05). Therefore, the blackcurrant extract exhibited moderate antibacterial activity against *E. coli*, *E. cloacae*, and *Yersinia enterocolitica*. Unfortunately, the extract showed no inhibitory effect on the tested Gram-positive bacteria, *Listeria monocytogenes* and *Enterococcus faecalis*. The *Salvia officinalis* extract showed the highest level of effectiveness against *Enterobacter cloacae*, with an inhibition zone of 17.17 ± 0.01 mm, indicating a moderate effect. A similar result was obtained for *Yersinia enterocolitica* (11.38 ± 0.01 mm), followed by *Escherichia coli* (11.26 ± 0.02 mm) and *Enterococcus faecalis* (10.97 ± 0.03 mm). Interestingly, the lowest inhibition zone was observed for *Listeria monocytogenes* (10.75 ± 0.04 mm), a Gram-positive bacterium. No effect was detected against *Salmonella enteritidis*, a Gram-negative bacterium.

**Figure 4:**
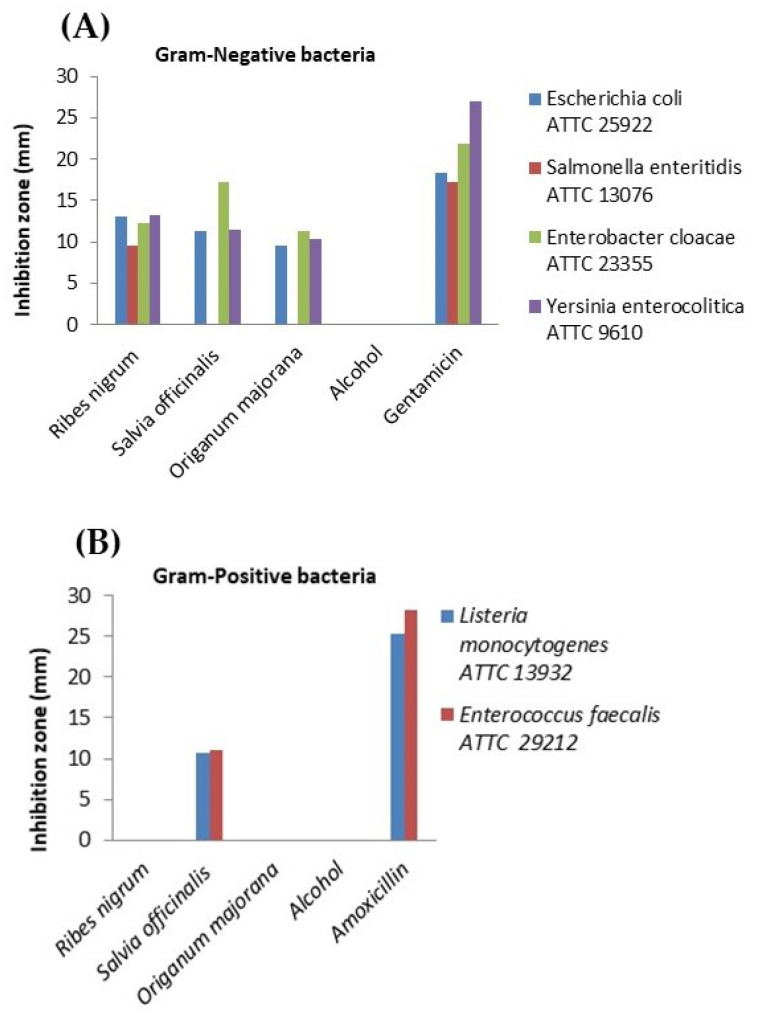
Antimicrobial activity of *Ribes nigrum*, *Origanum majorana,* and *Salvia officinalis* extracts by disk diffusion method against digestive bacteria. Controls used: gentamicin for Gram-negative bacteria (**A**); amoxicillin for Gram-positive bacteria (**B**).

The *Origanum majorana* (marjoram) extract also demonstrated antimicrobial activity. The largest inhibition zone was recorded for *Enterobacter cloacae* (11.33 ± 0.02 mm), followed by *Yersinia enterocolitica* (10.28 ± 0.00 mm), both Gram-negative bacteria. The smallest inhibition zone was observed for *Escherichia coli* (9.54 ± 0.04 mm). The extract showed no effect on any of the tested Gram-positive bacteria, nor on *Salmonella enteritidis*, a Gram-negative bacterium. All results are illustrated graphically in Figure 4.

### 2.3. Antimicrobial Activity by the Broth Microdilution Method

The minimum inhibitory concentration (MIC) and minimum bactericidal concentration (MBC) values for each tested bacterial strain are presented in Table 3.

Ribes nigrum exhibited the strongest antimicrobial effect against Yersinia enterocolitica (MIC 0.002 µg/µL; MBC 0.001 µg/µL), which was notably lower than the MIC values observed for other strains. The next effective inhibitory concentration, 0.018 μg/μL, was active against the Gram-negative *Enterobacter cloacae*. A concentration of 0.038 μg/μL inhibited the growth of both Gram-negative (*Salmonella enteritidis*, *Escherichia coli*) and Gram-positive (*Listeria monocytogenes*, *Enterococcus faecalis*) bacterial strains. Notably, the concentration of 0.038 μg/μL was also the first dose at which both inhibitory and bactericidal activity was recorded, specifically against *Salmonella enteritidis* and *Listeria monocytogenes*. The greatest bactericidal efficacy was observed against *Yersinia enterocolitica* at a remarkably low concentration of 0.001 μg/μL. The next bactericidal concentration, 0.038 μg/μL, was effective against both Gram-negative (*Salmonella enteritidis*, *Enterobacter cloacae*) and Gram-positive (*Listeria monocytogenes*) bacteria. The highest bactericidal concentration required was 0.082 μg/μL, which demonstrated activity against Gram-negative *Escherichia coli* and Gram-positive *Enterococcus faecalis*. Furthermore, based on the calculated MIC index values, which ranged between 0.5 and 2, the *Ribes nigrum* extract exhibited a bactericidal effect against all bacterial strains analyzed in this study.

The antimicrobial potential of *Salvia officinalis* extract was evaluated against several Gram-positive and Gram-negative bacterial strains. The maximum inhibitory effect was observed against the Gram-positive *Enterococcus faecalis* at a dose of 0.013 μg/μL. Notably, the Gram-negative *Salmonella enteritidis*, *Yersinia enterocolitica*, and the Gram-positive *Listeria monocytogenes* were inhibited at 0.027 μg/μL, while all remaining bacterial strains were inhibited at a 0.056 μg/μL concentration of the extract. Furthermore, the minimum concentration of *S. officinalis* extract that demonstrated both inhibitory and bactericidal effects was 0.056 μg/μL against *Enterobacter cloacae*, and 0.027 μg/μL against *Yersinia enterocolitica*. The lowest bactericidal concentration observed was 0.027 μg/μL for *Yersinia enterocolitica*, followed by 0.056 μg/μL for *Enterobacter cloacae*, *Listeria monocytogenes*, and *Enterococcus faecalis*. In contrast, the highest bactericidal dose, 0.121 μg/μL, was required to affect the Gram-negative strains *Escherichia coli* and *Salmonella enteritidis*. According to the minimum inhibitory concentration (MIC) index values, *Salvia officinalis* extract exhibited a bactericidal effect against all bacterial strains included in the study.

Similarly, the antimicrobial activity of *Origanum majorana* extract was assessed. The lowest inhibitory concentration was recorded at 0.026 μg/μL against Gram-negative *Yersinia enterocolitica* and Gram-positive *Enterococcus faecalis*. All remaining bacterial strains showed growth inhibition at a concentration of 0.054 μg/μL. The concentrations that exhibited both inhibitory and bactericidal effects were 0.026 μg/μL for *Yersinia enterocolitica* and 0.054 μg/μL for *Enterobacter cloacae*. The lowest bactericidal dose, 0.026 μg/μL, was effective against *Yersinia enterocolitica*. The next lowest bactericidal concentration, 0.054 μg/μL, was active against both *Salmonella enteritidis* and *Enterobacter cloacae*. The Gram-positive bacterial strains in this study were susceptible to a bactericidal concentration of 0.116 μg/μL, while the highest bactericidal dose, 0.203 μg/μL, was required for *Escherichia coli*. Based on the MIC index values, *Origanum majorana* extract also demonstrated bactericidal activity against all tested bacterial strains.

### 2.4. Statistical Analysis

The Kruskal–Wallis test revealed statistically significant differences (*p* < 0.05) among all tested combinations. Post hoc Dunn’s test showed that the only significant pairwise comparison was between Gentamicin and *Origanum majorana*. In contrast, no statistically significant differences were recorded among the plant extracts for any Gram-negative strains (*p* > 0.05), suggesting a similar level of antimicrobial activity. Similarly, no significant differences were found between the plant extracts and amoxicillin in Gram-positive strains, as inhibition by most extracts was absent or considerably lower than the antibiotic controls.

## 3. Discussion

The primary aim of this study was to evaluate the in vitro antimicrobial activity of *Ribes nigrum* fruit extract, *Origanum majorana*, and *Salvia officinalis* extracts against pathogenic gastrointestinal bacteria in companion animals.

The phytochemical composition of *Ribes nigrum*, *Salvia officinalis*, and *Origanum majorana* was assessed by quantifying the total phenolic and flavonoid content of their ethanolic extracts. Among them, *Salvia officinalis* had the richest polyphenolic profile (8509.457 µg/g), with *Origanum majorana* showing a slightly lower but comparable level (8140.996 µg/g). In contrast, *Ribes nigrum* demonstrated a lower concentration of phenolic compounds, measuring 5776.616 µg/g.

The lower polyphenol content in our *Origanum majorana* extract likely stems from several factors. Specific geographical and climatic conditions at the cultivation site impact polyphenol biosynthesis [13]. The plant’s genotype, even within the same species, can cause significant variations. The harvest time and specific plant part collected (e.g., flowering stage vs. young leaves) are also crucial [14]. Furthermore, post-harvest handling and storage can lead to polyphenol degradation. Differences in extraction methods and minor variations in analytical techniques can also influence the results [15,16]. We anticipate that the cultivation environment and genetic variations are the primary contributors. We will emphasize that such variations are common in natural product research, highlighting the importance of detailed reporting on plant sourcing and experimental conditions for accurate comparisons.

For *Salvia officinalis* and *Origanum majorana* extracts, our polyphenol levels, while exceeding some reports (e.g., Mokhtari et al. [17] for *S. officinalis*), were lower than others (e.g., Bendaas et al. [4] or A. El-Wardany et al. [7] for *O. majorana*). Key factors influencing these discrepancies include the extraction solvent choice, the specific plant part used, and variations in analytical techniques (e.g., Folin–Ciocalteu versus more specific methods like HPLC-DAD-ESI-MS), which can significantly alter the yield and profile of the extracted compounds.

Our Ribes nigrum total polyphenol content (5776.616 µg/g) was higher than some reported values (e.g., Rachtan-Janicka et al. [18] with 1891.5 µg/g from organic fruits) and comparable to others (Kikas et al.’s [19] 2.9–6.34 mg/g). However, we note that Paunović et al. [20] found significantly higher concentrations (15.55 to 16.94 mg GAE/g). In contrast, Kierońska et al. [21] reported a significantly lower polyphenol content of only 0.02385 mg/g (equivalent to 2.385 mg GAE/100 g). These variations likely stem from differences in cultivar, geographical origin, harvest time, and especially extraction methodologies (e.g., solvent type, duration, temperature) across studies.

In the present study, the *Salvia officinalis* extract exhibited a total polyphenol content of 8509.457 μg/g (equivalent to 8.509 mg/g), a significantly higher value compared to those previously reported in the literature. This notable difference highlights the variability inherent in polyphenol quantification across studies. For instance, Mokhtari et al. [17] determined a content of 6.43 mg GAE/g, while Mocan et al. [22] reported 4937.6 ± 293 μg/g of dry extract, corresponding to 4.9376 ± 0.293 mg/g, and a total polyphenol content of 65.02 ± 2.44 mg GAE/g. However, our results indicate a significantly lower polyphenol content when compared to other studies, such as that of Bendaas et al. [4], which reported a level of 234.80 mg GAE/g extract, Boufadi et al. [23] with 221.08 ± 2.36 mg GAE/g, or Svydenko [24], in which values ranged from 24.52 to 95.62 mg GAE/g.

Similarly, in the case of the ethanolic extract of *Origanum majorana*, the total polyphenol content determined in our study, 8140.996 μg/g (8.141 mg/g), proved to be considerably lower than the values reported in the literature. For example, A. El-Wardany et al. [7] reported a content of 900.52 mg GAE/g of dry extract, while Lahreche et al. [25] indicated a concentration of 164.96 ± 4.61 mg GAE/g. However, in the study conducted by AlJuhaimi et al. [15], the methanolic extract of *Origanum majorana* exhibited a total polyphenol content ranging from 259.17 mg GAE/100 g in fresh plant material to 1264.17 mg GAE/100 g in oven-dried plant material. The value obtained in our study (8140.996 μg GAE/g) falls within this range, suggesting a relative consistency with the literature data despite differences in extraction methods. Additionally, the essential oil of *Origanum majorana* was reported to have a total polyphenol content ranging between 11.60 and 188.67 μg GAE/mL, according to the findings of Öner et al. [26], underscoring the impact of extract type on measured polyphenol levels.

In addition to measuring overall polyphenol levels, a focused analysis of the key phenolic compounds was performed to enhance understanding of the extracts’ possible health-related properties. From a quantitative standpoint, 2,3-dihydroxybenzoic acid was the predominant phenolic compound identified in *Ribes nigrum* fruits in our study, followed by quercetin-rutinoside (rutin) and myricetin-glucoside. However, it is important to note that these results differ from several previous investigations, underscoring the complexity of phenolic profiles in blackcurrant fruits. These findings contrast with those reported by Rachtan-Janicka et al. [18], who identified delphinidin-3-O-rutinoside as the major compound, along with cyanidin derivatives (both rutinoside and glucoside forms) as secondary components. Similarly, in a study conducted by Kierońska et al. [21], blackcurrant fruits were characterized by a high content of gallic acid, catechin, and chlorogenic acid, highlighting the variability in phenolic composition that depends on cultivar, cultivation conditions, or the analytical method employed.

A total of 18 compounds were identified in the ethanolic extracts of both *Origanum majorana* and *Salvia officinalis*, classified into four distinct phytochemical groups. Among these, special attention was given to key phenolic compounds due to their known biological relevance. Although rosmarinic acid was the predominant compound in both species, *Salvia officinalis* (4157.949 μg/g = 4.158 mg/g) contained it in a slightly higher concentration than *Origanum majorana* (3972.428 μg/g = 3.972 mg/g). Our results are supported by the previous literature. For instance, a study conducted by A. El-Wardany et al. [7] reported rosmarinic acid as the primary polyphenol in *Origanum majorana* extract, with a concentration of 16,066.28 μg/g (16.06 mg/g). Similarly, Boufadi et al. [23] used HPLC analysis to identify rosmarinic acid as the major phenolic compound in the ethanolic extracts of *Salvia officinalis*, with a concentration of 7.85 mg/g.

However, variations in compound prevalence are also evident in the literature. In contrast, Mokhtari et al. [17] found that rosmarinic acid ranked only third in abundance in *Salvia* extract, being surpassed by apigenin and apigenin-7-glucoside. This variation highlights the importance of the extraction method and plant origin. In our study, apigenin was the fourth most abundant compound, with concentrations of 436.831 μg/g in *Salvia officinalis* and 498.117 μg/g in *Origanum majorana* extracts.

Moreover, the study reported high levels of chlorogenic acid (5331.02 μg/g) in *Origanum majorana* extract, whereas our results showed a significantly lower concentration of only 56.112 μg/g [7]. In contrast to these findings, chlorogenic acid was not detected in *Salvia officinalis* extract in the study by Mocan et al. [22], while in our research, a concentration of 124.741 μg/g was identified.

Additional inconsistencies were observed in the case of rutin. Interestingly, rutin was not detected in our *Origanum majorana* extract, whereas El-Wardany et al. [7] reported a concentration of 90.08 μg/g. In comparison, our *Salvia officinalis* extract contained 96.077 μg/g of rutin, a value lower than that reported by Mocan et al. [22], who found 1357.9 ± 34.4 μg/g.

One of the most striking differences in our findings relates to ferulic acid. Notably, unlike most of the data reported in the literature, our study revealed ferulic acid as the second most abundant phenolic compound, with concentrations of 1387.083 μg/g in *Origanum majorana* and 1164.369 μg/g in *Salvia officinalis*. By contrast, one study identified only 5.28 μg/g of ferulic acid in *Origanum majorana* extract [7]. On the other hand, a study reported a higher concentration in *Salvia officinalis* extract, 3.58 mg/g (3580 μg/g), which exceeds the levels found in our ethanolic extract [23]. Mokhtari et al. [17] reported that ferulic acid accounted for only 1.12% of the total compounds in the methanolic extract of *Salvia officinalis.*

Given the high polyphenolic content observed, it is essential to evaluate how these compounds contribute to the antimicrobial effects recorded in vitro.

The *Ribes nigrum* extract demonstrated notable antimicrobial efficacy, with a minimum inhibitory concentration (MIC) of 0.002 μg/μL against the Gram-negative bacterium *Yersinia enterocolitica*. This pronounced sensitivity suggests a potent interaction between the extract’s bioactive constituents and specific Gram-negative bacterial targets. Additionally, a concentration of 0.038 μg/μL was sufficient to inhibit the growth of other Gram-negative strains, including *Salmonella enteritidis* and *Escherichia coli*. The results obtained in this study highlight the antimicrobial activity of *Ribes nigrum* fruit extract, including efficacy against *Escherichia coli*. However, when compared to previous studies examining extracts from different plant parts, divergent results become apparent. For instance, Babayan and Sahakyan [27] reported that leaf extracts of *R. nigrum* lacked significant antimicrobial activity against *E. coli* ATCC 25922, despite their high polyphenol content (167.15 ± 7.29 mg GAE/g extract). In the present study, the total polyphenol content was higher in leaf extracts of *Ribes nigrum* compared to our fruit extracts, which contradicts the results of Paunović and Mašković [28], who reported that fruit extracts contain 1.95 to 4.29 times more phenolic compounds than leaf extracts. This discrepancy in biological activity may, in part, reflect compositional differences linked to the plant part used. Building upon the broad-spectrum activity demonstrated by *Ribes nigrum*, the *Salvia officinalis* extract similarly showed consistent antimicrobial effects across all tested bacterial strains. The highest effectiveness was observed against *Enterobacter cloacae*, with an inhibition zone of 17.17 ± 0.01 mm, indicating a moderate effect. To provide a broader perspective on the antimicrobial range, results against other strains were also evaluated. Comparable activity was recorded for *Yersinia enterocolitica* (11.38 ± 0.01 mm), followed by *Escherichia coli* (11.26 ± 0.02 mm) and *Enterococcus faecalis* (10.97 ± 0.03 mm). These results are consistent with those reported by Bendaas et al. [4], who observed an inhibition diameter of 15.66 ± 0.66 mm against *Escherichia coli*. In contrast, our study found a slightly smaller inhibition zone (11.26 ± 0.02 mm). Similar findings were reported by AlFadhly [8] and Mokhtari et al. [17], who noted an inhibition zone of 16.76 mm against *E. coli*. However, regarding *Enterococcus faecalis*, the extract tested in our study showed more potent antimicrobial activity (10.97 ± 0.03 mm) compared to that reported by Bendaas et al. [4] (7.33 ± 0.33 mm). Compared to the previous literature, the microdilution assay results in our study revealed superior antimicrobial activity. The strongest inhibition was observed against the Gram-positive *Enterococcus faecalis* at a concentration of just 0.013 μg/μL, whereas Bendaas et al. [4] required a much higher concentration of 100 μg/μL for the same effect. In the case of Gram-negative strains, findings were more variable. For the Gram-negative *Salmonella enteritidis*, inhibition occurred at 0.027 μg/μL in our study, a value very close to that reported (0.025 μg/μL) [4]. In contrast, *Escherichia coli* was inhibited at 0.056 μg/μL in our research, compared to 0.1 μg/μL in the reference study [4]. These findings suggest that, while the efficacy against *Salmonella enteritidis* is comparable, the extract tested in our study exhibited superior antimicrobial efficiency against *Enterococcus faecalis* and *Escherichia coli*, requiring significantly lower concentrations to inhibit bacterial growth. This enhanced effectiveness is also supported by another study, which reported much higher MIC and MBC values for *Salvia officinalis* against *E. coli* and *Salmonella enteritidis*—2500 μg/mL and 2500 μg/mL for *E. coli*, and 2500 μg/mL and 1250 μg/mL for *Salmonella enteritidis* [29]. Additionally, a study conducted by Mocan et al. [22] found that the ethanolic extract of *Salvia officinalis* showed antimicrobial activity against *Listeria monocytogenes*, with a minimum inhibitory concentration (MIC) of 0.18 mg/mL and a minimum bactericidal concentration (MBC) of 0.36 mg/mL. In comparison, our extract demonstrated higher antimicrobial efficacy, with an MIC of 0.027 μg/μL (equivalent to 0.027 mg/mL) and an MBC of 0.056 μg/μL (0.056 mg/mL).

The *Origanum majorana* extract demonstrated a promising inhibitory effect on bacterial growth. To better understand the extent of this activity, both inhibition zones and MIC values were analyzed. Based on MIC values, the extract exhibited bactericidal activity against all tested bacterial strains. In the case of *Escherichia coli*, *O. majorana* extract produced an inhibition zone of 9.54 mm, which is lower than the 17 mm reported in the literature [7]. This discrepancy may be attributed to differences in extraction methods or compound stability. Furthermore, according to a 2025 study, the essential oil of *Origanum majorana* inhibited *E. coli* growth with a zone of 20 mm, suggesting that volatile compounds may exert stronger antimicrobial effects compared to ethanolic extracts [26]. Regarding *Listeria monocytogenes*, contrary to the findings of a previous study that reported an inhibition zone of 14 mm, no detectable antimicrobial activity was observed in our study against this pathogen [7]. To further assess the antimicrobial potential, MIC values were considered as a more precise metric. The minimum inhibitory concentration (MIC) of the ethanolic extract obtained in our study was 0.026 μg/μL against both *Yersinia enterocolitica* (Gram-negative) and *Enterococcus faecalis* (Gram-positive), while growth inhibition of *E. coli* was observed at a concentration of 0.054 μg/μL. In comparison, previous studies using essential oil preparations have reported different levels of activity. By comparison, the study conducted by Öner et al. [26] reported MIC values of 0.016 mg/mL for *E. coli* and 0.032 mg/mL for *Enterococcus faecalis* when testing *O. majorana* essential oil. These results suggest notable antimicrobial efficacy, although influenced by the different chemical natures of the extracts used, namely, volatile oil versus ethanolic extract.

To sum up, while the findings are encouraging, further investigation into the underlying molecular mechanisms is essential to fully unlock the therapeutic potential of the plants analyzed.

## 4. Materials and Methods

### 4.1. Reagents and Chemicals

HPLC: Ethanol 96% (Sigma Aldrich, Darmstadt, Germany) was used for the preparation of the extracts. HPLC-grade acetonitrile was purchased from Merck (Darmstadt, Germany), and ultrapure water was purified using the Direct-Q UV system from Millipore (Darmstadt, Germany). Standard gallic and chlorogenic acids (98% HPLC purity), as well as luteolin, rutin, and cyanidin (99% HPLC purity), were purchased from Sigma Aldrich (Darmstadt, Germany).

Microbiology: The standard and special culture media were acquired from BioMaxima S.A., Lublin, Poland, and Bio-Rad Laboratories Inc., Hercules, CA, USA, respectively. Mueller–Hinton agar and broth were supplied by Merck, Darmstadt, Germany, while Liofilchem, Teramo, Italy, provided the antibiotic disks. The reference bacterial strains were acquired from BioMaxima S.A., Lublin, Poland. The remaining substances were bought from Sigma Aldrich, Darmstadt, Germany.

### 4.2. Plants and Fruit Material

The dried and ground plants were sourced from local distributors specializing in phytotherapeutic products, who confirm that these plants originate from their own cultivation. This cultivation is conducted in carefully selected areas with optimal soil quality and favorable climatic conditions, ensuring the plants are grown naturally, without chemical treatments. The plants are harvested at the optimal time when the content of active compounds is at its highest. After a rigorous selection process, the plants are dried using a customized protocol. They are then stored under controlled temperature and humidity conditions, labeled, and organized into batches. Each batch undergoes a comprehensive set of analyses conducted over a 24 h period to ensure the quality, purity, and therapeutic efficacy of the final product.

The fruits, acquired in freeze-dried form and unground, undergo a delicate preservation process. Freeze-drying involves the rapid freezing of fresh fruits, followed by dehydration under a vacuum at controlled temperatures. This process removes water by sublimation, without melting, preserving the aroma, color, vitamins, and nutritional value of the fruits.

### 4.3. Plants and Fruit Extract Preparation

Initially, 1 g of ground plant material (sage or marjoram) was mixed with 60 mL of 96% ethanol and stirred for one hour at room temperature using a magnetic stirrer (VELP Scientifica, Usmate Velate, Italy).

We selected 96% ethanol for our extractions due to its effectiveness as a relatively non-toxic and eco-friendly solvent. It efficiently extracts a broad spectrum of phenolic compounds, including flavonoids, phenolic acids, and anthocyanins, all abundant in *Origanum majorana*, *Salvia officinalis*, and *Ribes nigrum*. Previous research on similar plant matrices consistently demonstrates ethanol’s success, often at concentrations from 70 to 96%. This strong precedent, combined with our goal of extracting a wide array of bioactive compounds, made 96% ethanol the optimal choice, aligning with established methodologies in natural product chemistry [30,31].

The mixture was then filtered, and the remaining residue was subjected to a second extraction with another 60 mL of ethanol, stirred for 30 min. After filtration, the residue was extracted once more with 30 mL of ethanol and stirred for 30 min. After the final filtration, all filtrate fractions were combined, resulting in 123 mL of marjoram extract and 135 mL of sage extract.

Similarly, blackcurrant fruits were ground into a fine powder and mixed with 30 mL of ethanol, then stirred for one hour. After filtration, the residue was re-extracted twice, each time with 20 mL of ethanol and stirred for 30 min. All liquid fractions from the three extraction steps were combined, resulting in 66 mL of extract.

To remove the ethanol, an evaporator (CONCENTRATOR plus; Eppendorf, Hamburg, Germany) was used, set at 45 °C. Subsequently, for polyphenol content analysis using HPLC-DAD-ESI-MS and for microbiological testing, the extracts were reconstituted in 10 mL of 70% ethanol, obtained by mixing 96% ethanol with distilled water.

### 4.4. Determination of Total Phenolic Content: High-Performance Liquid Chromatography–Diode Array Detection–Electro-Spray Ionization Mass Spectrometry (HPLC-DAD-ESI-MS) for Polyphenols Profile—Chromatography Analysis

An HPLC Agilent 1200 system equipped with a G1311A Quaternary Pump, G1322A degasser, G1329A autosampler, UV–Vis detector with G1315D photo-diode array (PDA) coupled with a mass detector, single quadrupole Agilent model 6110 (Agilent Technologies, Santa Clara, CA, USA). The separation of compounds was performed on a Kinetex XB C18 column, 4.6 × 150 mm, 5 μm (Phenomenex, Torrance, CA, USA). Mobile phases (A) water and acid acetic (0.1%), respective (B) acetonitrile and acetic acid (0.1%) were used for the following gradient: 0 min, 5% B; 0–2 min, 5% B; 2–18 min, 5–40% B; 18–20 min, 40–90% B; 20–24 min, 90% B; 24–25 min, 90–5% B; 25–30 min, 5% B. The chromatogram was recorded at 280, 340, and 520 nm wavelength for 30 min, at 250 °C, at a flow of 0.5 mL/min. For MS analysis, the full scan ESI positive ionization mode was used in the following conditions: capillary tension 3000 V, temperature 3500 °C, nitrogen debit 7 L/min, energy fragmentor 100 V, and *m*/*z* 120–1200. The data acquisition and interpretation were completed using the Agilent ChemStation software, version Rev B.02.01-SR2.

The identification of phenolic compounds was made based on their retention times and absorption spectra in comparison to the standards dissolved in methanol and based on the literature and database Phenol-Explorer (http:/phenol-explorer.eu). Quantification of phenolic compounds was completed by using the calibration curves of 1-Gallic acid (R2 = 0.9978), LOD = 0.35 μg/mL, LOQ = 1.05 μg/mL; 2-Chlorogenic acid (R2 = 0.9937), LOD = 0.41 μg/mL, LOQ = 1.64 μg/mL; 3-Rutine (R2 = 0.9981), LOD = 0.21 μg/mL, LOQ = 0.84 μg/mL; 4-Luteoline (R2 = 0.9972), LOD = 0.38 μg/mL, LOQ = 1.46 μg/mL si 5-Cyanidine (R2 = 0.9951), LOD = 0.36 μg/mL, LOQ = 1.44 μg/mL. The hydroxycinammic acids were quantified as chlorogenic equivalents, hydroxybenzoic acids as gallic acid equivalents, flavones as luteoline equivalents, flavanols as rutine equivalents, respectively, and anthocyanins as cyanidins equivalents. Before chromatographic analysis, the samples were passed through Chromafil Xtra nylon (Merck, Darmstadt, Germany) 0.45 µm filters, and 20 μL was injected into HPLC.

### 4.5. Antimicrobial Activity of Investigated Plants and Fruit Extracts

This study aimed to test the antimicrobial activity of three plant extracts, blackcurrant, sage, and marjoram, using two testing methods—the agar diffusion method and the determination of the minimum inhibitory concentration (MIC). For both methods, the following standardized bacterial strains were used: *Escherichia coli* ATCC 25922, *Salmonella enteritidis* ATCC 13076, *Enterobacter cloacae* ATCC 23355, *Yersinia enterocolitica* ATCC 9610, *Listeria monocytogenes* ATCC 13932, and *Enterococcus faecalis* ATCC 29212.

#### 4.5.1. Testing the Antibacterial Activity of Extracts Using the Diffusion Method

The antibacterial activity of the extracts was tested on Mueller–Hinton agar plates (Merck, Darmstadt, Germany) following the protocol below: the reference bacterial strains were subcultured on Columbia agar supplemented with 5% sheep blood and incubated for 24 h at 37 °C in aerobic conditions to obtain fresh cultures. The following day, bacterial suspensions were prepared in sterile physiological saline for each strain, maintaining a turbidity standard of 0.5 on the McFarland scale, which was verified using an optical densitometer (Biosan, Riga, Latvia).

The agar plates were inoculated with the bacterial suspensions following the EUCAST protocol for susceptibility testing [32]. Briefly, using a sterile cotton swab soaked in the bacterial suspension, the surface of the agar was streaked in three directions to ensure uniform coverage.

A sterilized metal punch was used to create four wells in the agar, each 6 mm in diameter, arranged in a radial pattern. Into each well, 60 µL of the following solutions were deposited: blackcurrant extract, sage extract, marjoram extract, and 70% ethanol (as solvent control). Additionally, for each reference strain, the activity of a synthetic antibiotic was also tested using commercial microdisks. Gentamicin (GEN 10 µg, Liofilchem, Teramo, Italy) was used for Gram-negative bacteria, while Amoxicillin (AX 10 µg, Liofilchem, Teramo, Italy) was used as a control for Gram-positive bacteria (Figure S1).

The plates were incubated in aerobic conditions for 24 h at 37 °C. The diameters of the inhibition zones were measured the next day using an electronic caliper. All samples were tested in duplicate.

#### 4.5.2. Determination of the Minimum Inhibitory Concentration (MIC)

To determine the MIC, sterile 96-well microtiter plates were used (microdilution method). Bacterial suspensions for each strain were prepared in sterile physiological saline at a concentration of 10^6^ CFU (colony-forming units)/mL. Starting from the first well in each row, with an initial 1/1 dilution (90 µL of the extract), serial two-fold dilutions of the tested extract were performed using the same volume of Mueller–Hinton broth (90 µL), continuing up to a 1/512 dilution. Then, 10 µL of the reference bacterial suspension was added to each well. Each row included a negative control (70% ethanol) and a positive control (Mueller–Hinton broth inoculated with bacterial suspension). The prepared plates were incubated at 37 °C for 24 h. Results were read the following day, with the minimum inhibitory concentration (MIC) defined as the lowest dilution where no bacterial growth (no visible pellet or turbidity) was observed. For each strain and extract, the minimum bactericidal concentration (MBC) was also assessed. The protocol involved inoculating Mueller–Hinton agar plates with 10 µL from each well where no bacterial growth was detected, followed by incubation at 37 °C for 24 h. The MIC index was calculated based on the MBC/MIC ratio to determine whether the extract exhibited a bactericidal effect (MBC/MIC ≤ 4) or a bacteriostatic effect (MBC/MIC > 4) on the tested strains. The plates were incubated in aerobic conditions for 24 h at 37 °C. All samples were tested in duplicate.

### 4.6. Statistical Analysis

Statistical analysis was conducted using SPSS (IBM SPSS Statistics, Version 26) and Microsoft Excel 2016. Comparative analysis of inhibition zone diameters was performed using the Kruskal–Wallis non-parametric test, followed by Dunn’s post hoc test for pairwise comparisons. Independent samples *t*-tests were applied where appropriate. A *p*-value < 0.05 was considered statistically significant.

## 5. Conclusions

The present study highlights the promising antibacterial potential of *Origanum majorana*, *Salvia officinalis*, and *Ribes nigrum* fruit ethanolic extracts as natural alternatives for managing digestive bacterial infections. All extracts exhibited bactericidal activity against both Gram-positive and Gram-negative bacterial strains, with *Ribes nigrum* demonstrating enhanced efficacy against Gram-negative strains and *Salvia officinalis* showing notable activity against Gram-positive strains. These effects are likely attributed to their elevated polyphenolic content, which may also potentiate the effectiveness of conventional antibiotics such as gentamicin and amoxicillin. Among the tested extracts, *Salvia officinalis* recorded the highest total polyphenol content, followed by *Origanum majorana* and *Ribes nigrum*. The potential integration of these plant-based extracts into therapeutic strategies could reduce the need for high-dose or frequent antibiotic use in both human and veterinary medicine, contributing to the broader goal of limiting antimicrobial resistance. Overall, this study highlights the antibacterial effects of these plant species and underscores their adjuvant role in antibiotic therapy.

Given the considerable variability observed in the phytochemical composition, future research should focus on a deeper examination of how genetic background and environmental conditions influence the biosynthesis of active compounds. Investigating a broader range of extraction techniques and solvent systems is equally important, along with efforts to standardize phytochemical extraction protocols to improve reproducibility across studies. Furthermore, in vivo studies are essential to better understand the pharmacokinetics, bioavailability, and therapeutic potential of the extracts, including their possible synergistic effects when combined with antibiotics. This includes investigations into extract toxicity, optimization of extraction methods using green technologies or alternative solvents, as well as molecular studies exploring antimicrobial mechanisms.

## Figures and Tables

**Table 1:** HPLC-DAD-ESI-MS analysis of phenolic compounds of *Ribes nigrum* extract.

Peak No.	Retention Time Rt (min)	UV λmax (nm)	[M + H]^+^ (*m*/*z*)	Compound	Subclass	Concentration (μg/g)
**1**	2.77	280	155	2,3-Dihydroxybenzoic acid	Hydroxybenzoic acid	978.266
**2**	10.08	530,280	465	Delphynidin-glucoside	Anthocyanin	144.331
**3**	10.44	530,280	611	Delphynidin-rutinoside	Anthocyanin	382.550
**4**	11.13	535,280	449	Cyanidin-glucoside	Anthocyanin	287.370
**5**	11.51	535,280	595	Cyanidin-rutinoside	Anthocyanin	165.124
**6**	13.79	350,260	433,287	Kaempferol-rhamnoside	Flavonol	228.990
**7**	13.98	360,255	627,303	Quercetin-diglucoside	Flavonol	448.036
**8**	14.35	360,260	481,319	Myricetin-glucoside	Flavonol	894.295
**9**	15.01	360,255	611,303	Quercetin-rutinoside (Rutin)	Flavonol	973.003
**10**	15.69	360,255	465,303	Quercetin-glucoside	Flavonol	371.926
**11**	16.05	360,255	449,303	Quercetin-rhamnoside	Flavonol	368.585
**12**	16.69	355,260	449,287	Kaempferol-glucoside	Flavonol	97.934
**13**	16.99	355,255	479,317	Isorhamnetin-glucoside	Flavonol	106.844
**14**	17.41	360,260	465,319	Myricetin-rhamnoside	Flavonol	77.514
**15**	18.26	360,260	319	Myricetin	Flavonol	102.760
**16**	20.87	360,255	303	Quercetin	Flavonol	117.982
**17**	23.10	355,260	287	Kaempferol	Flavonol	31.106
				**Total phenolics**		**5776.616**

**Table 2:** HPLC-DAD-ESI-MS analysis of phenolic compounds of *Salvia officinalis* and *Origanum majorana* extracts.

Peak No.	Retention Time Rt (min)	λmax (nm)	[M + H]^+^ (*m*/*z*)	Phenolic Compound	Subclass	Concentration (μg/g)	
						Marjoram	Sage
**1**	2.77	280	155	2,3-Dihydroxybenzoic acid	Hydroxybenzoic acid	585.689	209.469
**2**	11.47	330	355	5-Caffeoylquinic acid (Chlorogenic acid)	Hydroxycinnamic acid	56.112	124.741
**3**	12.82	340,250	595,287	Luteolin-rutinoside	Flavone	101.789	173.241
**4**	13.97	345,250	433,271	Apigenin-glucoside	Flavone	10.004	47.038
**5**	14.32	345,250	475,271	Apigenin-acetyl-glucoside	Flavone	10.295	46.892
**6**	15.07	356,255	611,303	Quercetin-rutinoside (Rutin)	Flavonol	NF	96.077
**7**	15.31	340,250	463,287	Luteolin-glucuronide	Flavone	235.109	282.017
**8**	15.65	340,250	449,287	Luteolin-glucoside	Flavone	215.648	308.594
**9**	16.01	356,257	465,303	Quercetin-glucoside (Isoquercitrin)	Flavonol	237.158	471.425
**10**	16.71	345,250	447,271	Apigenin-glucuronide	Flavone	137.079	122.411
**11**	17.15	331	361	Rosmarinic acid	Hydroxycinnamic acid	3972.428	4157.949
**12**	17.63	330	195	Ferulic acid	Hydroxycinnamic acid	1387.083	1164.369
**13**	20.67	340,250	287	Luteolin	Flavone	61.706	279.113
**14**	21.11	356,257	303	Quercetin	Flavonol	105.359	NF
**15**	21.59	340	345	Rosmadial	Terpene	373.221	64.756
**16**	22.76	345,250	271	Apigenin	Flavone	498.117	436.831
**17**	22.31	340	347	Rosmanol	Terpene	106.291	338.221
**18**	24.30	340	333	Carnosic acid	Terpene	47.909	186.312
				**Total phenolics**		**8140.996**	**8509.457**

NF: not found.

**Table 3:** Antimicrobial activity *of Ribes nigrum*, *Origanum majorana,* and *Salvia officinalis* extracts by broth microdilution method against digestive bacteria.

MIC Index MIC (μg GAE/μL)/MBC (μg GAE/μL)			
**Bacterial Isolates**	*Ribes nigrum*	*Salvia officinalis*	*Origanum majorana*
*Escherichia coli*	**2** 0.038/0.082	**2** 0.056/0.121	**4** 0.054/0.203
*Salmonella enteritidis*	**1** 0.038/0.038	**4** 0.027/0.121	**1** 0.054/0.054
*Enterobacter cloacae*	**2** 0.018/0.038	**1** 0.056/0.056	**1** 0.054/0.054
*Yersinia enterocolitica*	**0.5** 0.002/0.001	**1** 0.027/0.027	**1** 0.026/0.026
*Listeria monocytogenes*	**1** 0.038/0.038	**2** 0.027/0.056	**2** 0.054/0.116
*Enterococcus faecalis*	**2** 0.038/0.082	**4** 0.013/0.056	**4** 0.026/0.116

Bold—MIC index value; ≤4 = bactericidal effect, >4 = bacteriostatic effect.

## Data Availability

The data presented in this study are available on request from the corresponding authors.

## Supplementary Materials

The following supporting information can be downloaded at: https://www.mdpi.com/article/10.3390/molecules30163341/s1, Figure S1: Agar well diffusion assay illustrating the antibacterial activity of plant extracts (*Ribes nigrum*—R.N., *Salvia officinalis*—S.O., and *Origanum majorana*—O.M.) against all tested bacterial strains: *Escherichia coli*, *Salmonella enteritidis*, *Enterobacter cloacae*, *Yersinia enterocolitica*, *Listeria monocytogenes* and *Enterococcus faecalis*.
