# Supplementary material for: Antibacterial Activity of Ethanolic Extracts of Origanum majorana, Salvia officinalis, and Ribes nigrum Against Digestive Pathogens: Polyphenolic Composition and In Vitro Assessment
Source: Molecules. 2025 Aug 11;30(16):3341. doi: 10.3390/molecules30163341 (PMC12388215; doi:10.3390/molecules30163341)
Supplement: Supplementary file 1 [file molecules-30-03341-s001.zip › molecules-3782191-supplementary.pdf]

Figure S1. Agar well diffusion assay illustrating the antibacterial activity of plant extracts (*Ribes nigrum*-R.N., *Salvia officinalis*-S.O., and *Origanum majorana*-O.M.) against all tested bacterial strains: *Escherichia coli*, *Salmonella enteritidis*, *Enterobacter cloacae, Yersinia enterocolitica*, *Listeria monocytogenes* and *Enterococcus faecalis*.

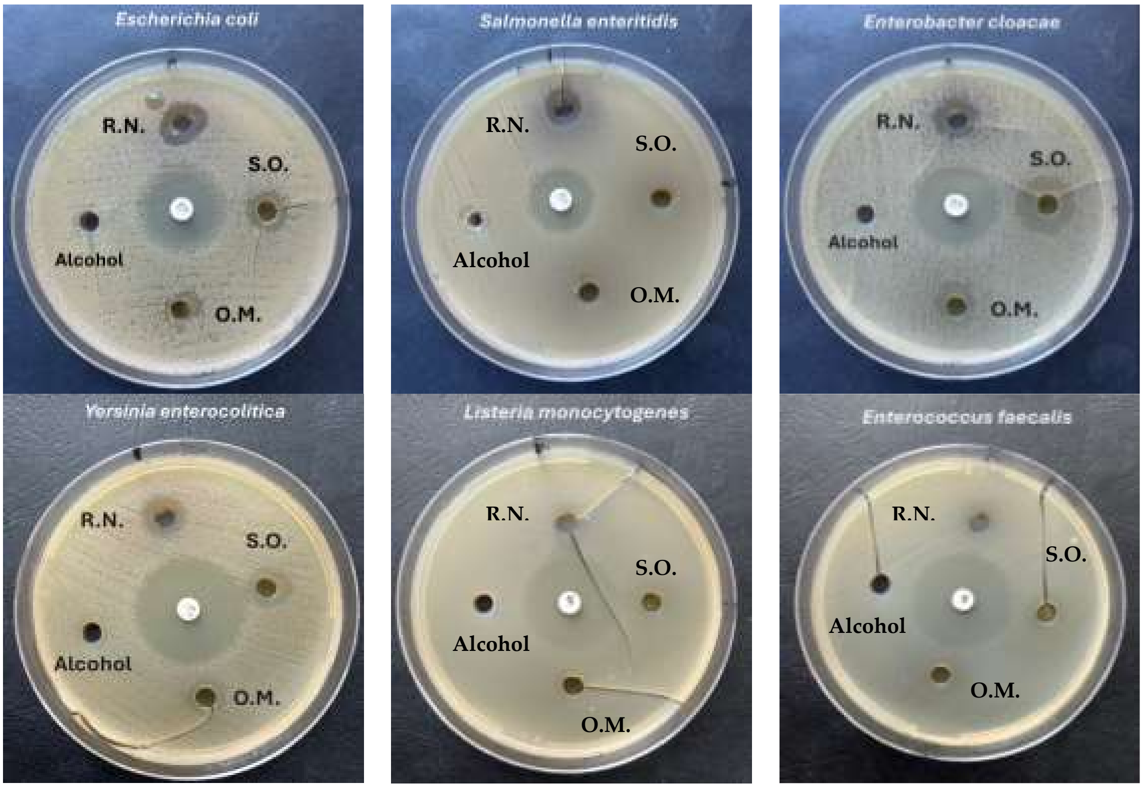

Each Petri dish shows inhibition zones surrounding the wells filled with plant extracts. The central well in each plate contains the specific antibiotic used as a positive control: gentamicin for Gram-negative bacteria and amoxicillin for Gram-positive strains. This figure supports the quantitative data presented by visualizing the extract efficacy across both Gram-negative and Gram-positive pathogens.
